# Supplementary material for: Reproducibility and FAIR Principles: The Case of a Segment Polarity Network Model
Source: ArXiv. 2023 Apr 18:arXiv:2304.08688v1. Preprint. [Version 1] (PMC10153292)
Supplement: Supplement 1 [file NIHPP2304.08688v1-supplement-1.pdf]

Supplemental Figures

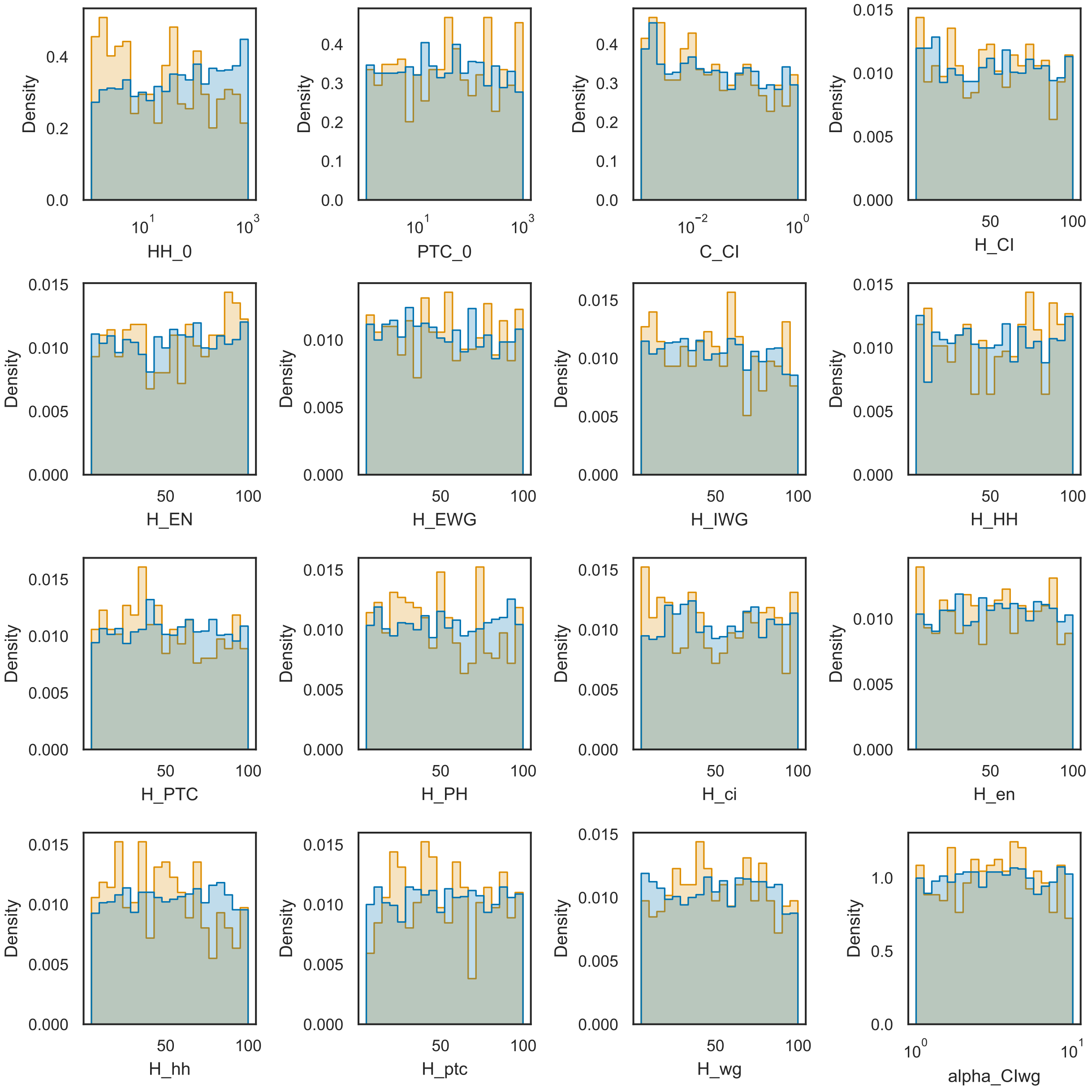

Figure S1: Distributions of parameter values that result in a single steady state (blue) and multiple steady states (orange).

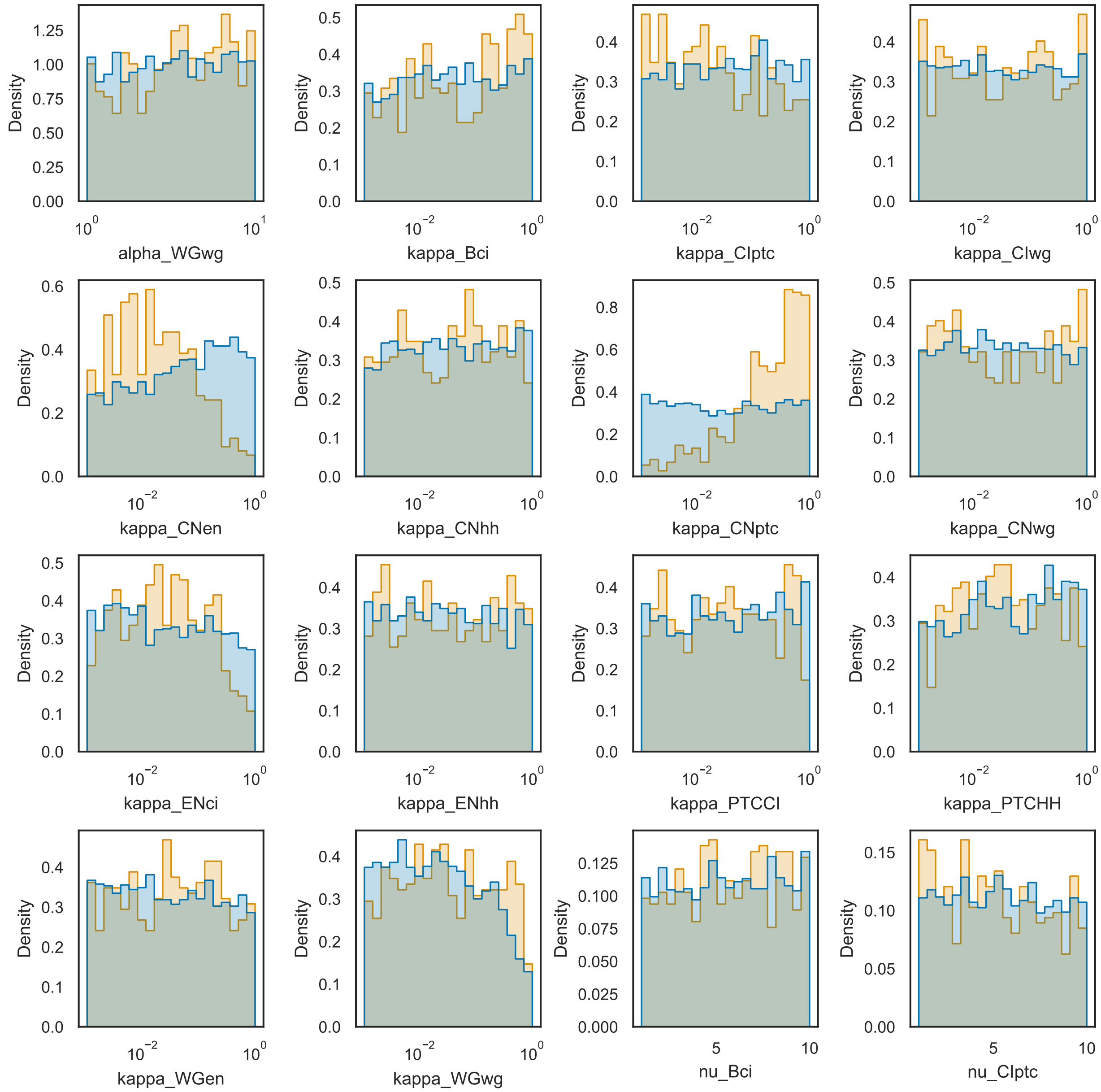

Figure S2: Distributions of parameter values that result in a single steady state (blue) and multiple steady states (orange).

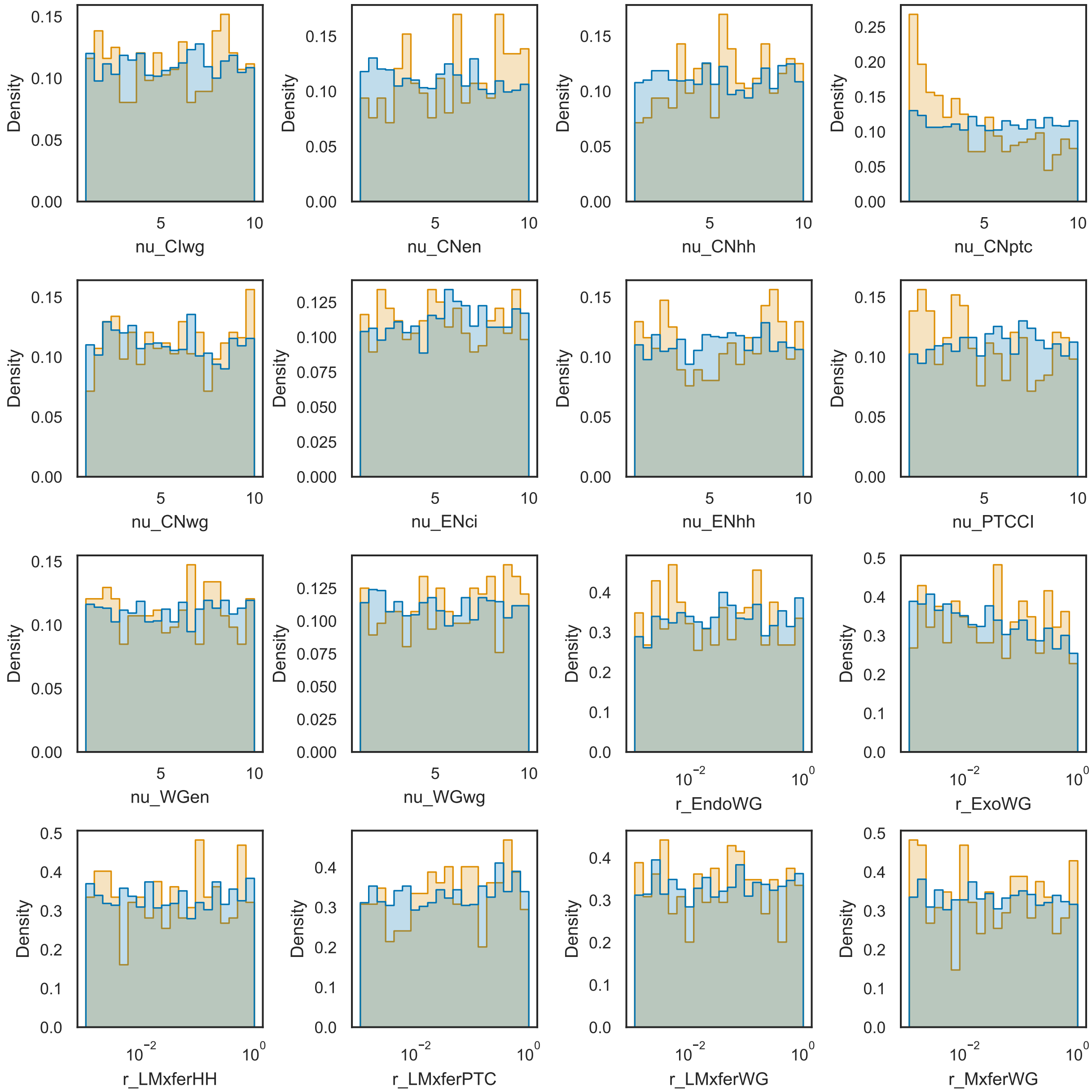

Figure S3: Distributions of parameter values that result in a single steady state (blue) and multiple steady states (orange).
